# Polycystic Ovarian Syndrome in Aging Women: An Observational Study

**DOI:** 10.7759/cureus.29776

**Published:** 2022-09-30

**Authors:** Mukta Agarwal, Sudwita Sinha, Pallavi Lohani, Ritu Singh, Simran Dureja

**Affiliations:** 1 Obstetrics and Gynecology, All India Institute of Medical Sciences, Patna, IND; 2 Community and Family Medicine, Madhubani Medical College, Patna, IND

**Keywords:** outcome, management, presentations, aging women, pcos

## Abstract

Background: Polycystic ovarian syndrome (PCOS) in aging women has not been extensively studied, although it is a very common disorder. Most of the research has been conducted on women of the reproductive age group, although PCOS is a disease occurring in all age groups. This study aims to provide an idea of the PCOS pattern prevalent in aging women presenting at a tertiary care center in Eastern India.

Methods: This was a prospective, single-center, observational cohort study conducted over three years, from October 2016 to September 2019. A total of 36 patients between 35 and 65 years of age were found to have PCOS according to Rotterdam’s criteria during this period. These patients were prospectively followed up to study their demographic characteristics, symptoms, management, and outcomes.

Results: A total of 80% of the women were below 40 years of age, while only 20% were above 40. In total, 70% of the women were overweight or obese. Infertility was seen in 75% of patients below 40 years of age and 28% of patients above 40. Among these, 23 (95%) conceived successfully after proper management. The main presenting complaints were infertility and menstrual irregularities. With aging, diabetes increased from 24%, in below 40, to 28%, in above 40; hypertension increased from 13% to 28%. The occurrence of impaired oral glucose tolerance test (OGTT) and impaired lipid profile increased with age: from 48% to 57% and 13% to 28%, respectively.

Conclusion: PCOS in aging women causes considerable morbidity and greatly affects the quality of life, although it is less understood. Further research in this field is very much needed to understand and appropriately manage the problems in aging women, to improve their quality of life.

## Introduction

Once referred to as the ‘medical black hole’, polycystic ovarian syndrome (PCOS) in aging women has not been extensively studied, although it is a very common disorder [1,2]. Previous studies available have been conducted mainly on women of reproductive age, with very few studies available on aging women [2].

PCOS, the most common hormonal, metabolic and reproductive endocrinopathy among women, with a prevalence of 5%-18%, has a lifelong adverse health impact, evidenced by decreased health-related quality of life [3-5]. It is characterized by the presence of oligoovulation or anovulation, hyperandrogenism, and polycystic ovarian morphology, and is associated with a high risk of obesity, insulin resistance, dyslipidaemia, impaired glucose tolerance, type 2 diabetes mellitus, metabolic syndrome, cardiovascular events, increased risk of psychological morbidity, asthma, migraine, obstructive sleep apnoea, endometrial carcinoma and mood disorders [3-5]. Three sets of proposed diagnostic criteria produced by the National Institutes of Health (NIH), Rotterdam, and Androgen Excess and PCOS (AE-PCOS) Society guidelines are available for defining PCOS [6,7]. Other mimicking conditions, such as hyperprolactinemia, hypercortisolaemia, thyroid disorders, and congenital adrenal hyperplasia, must be excluded before a diagnosis of PCOS is made [5]. Rotterdam criteria require at least two of the following for a diagnosis of PCOS: oligo-anovulation (OA), hyperandrogenism (HA), or the presence of polycystic ovarian morphology [7]. PCOS typically manifests during the early reproductive years with a heterogenous clinical expression, including menstrual dysfunction, infertility, and signs of androgen excess, that varies between women [5]. No diagnostic criteria are currently available for PCOS in aging women [8]. A presumptive diagnosis of PCOS in aging women can be made based on a long-term history of PCOS in reproductive age, hyperandrogenism, a chronic history of irregular menstrual cycles, and/or polycystic ovarian morphology on ultrasonography (USG). PCOS can be difficult to diagnose in aging women since aging leads to alterations in all three diagnostic criteria [8].

Lifestyle management and modifications are advised for primary prevention, targeting dyslipidaemia and glucose abnormalities, and if necessary, metformin and treatments for dyslipidaemia are added [8]. The reproductive life span in PCOS women extends beyond that of non-PCOS women due to higher adrenal and ovarian androgen levels [2]. Increased waist circumference and BMI can impact body image negatively, affect self-esteem, and increase depression risk [2]. For some women who struggle with infertility, a higher likelihood of pregnancy may be there, as they get older. Dietitians can provide nutrition counselling and education to make positive changes in eating habits and subsequently reduce the risk of disease and improve health. A loss of 5%-10% of the total body weight improves both reproductive and metabolic parameters [2].

Long-term studies in aging PCOS women are, however, scarce and the hormonal and metabolic changes associated with the late reproductive years and beyond menopause are poorly understood [3]. Little is known about the impact on health status in aging women with PCOS. The syndrome often remains underrepresented in patients’ records and national registers, as it is usually underdiagnosed, which, therefore, limits capturing comorbidities. The available data on aging PCOS women are inconclusive, as most of the previous studies have been retrospective and of a cross-sectional design with a small sample size, incomplete diagnosis, inadequate measurements, and inclusion of younger PCOS women. Hence, future research with large prospective studies is needed with adequately sized and well-characterized PCOS populations.

This study aims to provide an idea of the prevalent PCOS pattern in aging women at a tertiary care center in Eastern India. It focuses on assessing morbidities, self-reported symptoms, management, and outcomes in aging women with PCOS.

## Materials and methods

Study design

The present study was a prospective single-centered observational cohort study conducted over three years from October 2016 to September 2019 in a tertiary care institute in Eastern India. It was conducted after approval by the Institute Ethics Committee, All India Institute of Medical Sciences (AIIMS), Patna (AIIMS/Pat/IEC/2016/110).

Inclusion/Exclusion Criteria

During the aforesaid period, all the patients above 35 years of age presenting to the gynecology outpatient department (AIIMS, Patna) with symptoms suggesting of PCOS were screened for any of the two features of PCOS according to Rotterdam’s criteria. The inclusion criteria included women presenting to the gynecology outpatient department meeting Rotterdam’s diagnostic criteria of PCOS, in the age group of 35-65 years, and consenting to participate in the study. Women below 35 or above 65 years of age, not meeting Rotterdam’s criteria, and not consenting were not included in the study.

Data Collection

During the aforesaid three-year period, a total of 95,130 patients attended the outpatient department of Obstetrics and Gynecology of which 38,526 patients had gynecological complaints while the rest were obstetric patients. Out of 38,526 gynecologic patients, 17,158 patients were above the age of 35 years. Of these, 857 patients presented with symptoms suggesting of PCOS and were screened for any of the two features of PCOS according to Rotterdam’s criteria. A total of 36 patients in the age group of 35-65 years were found to have PCOS according to Rotterdam’s criteria during the defined period. In post-menopausal women in whom menstrual irregularity was not a presenting symptom, a long-term history of PCOS in reproductive age, a chronic history of menstrual irregularities during the pre-menopausal period, features of hyperandrogenism, presence of endometrial hyperplasia or carcinoma or polycystic ovarian morphology on USG were taken as criteria for presumptive diagnosis of PCOS. All the participants furnished informed consent for the use of their collected data for scientific purposes. Clinical history and general and systemic examination were performed for all patients and their clinical parameters such as body mass index (BMI), parity, clinical presentation (infertility, menstrual irregularities, hirsutism), and the presence of ultrasonography criteria for diagnosing PCOS were observed. A note was also made of the presence of associated comorbidities like type 2 diabetes mellitus, hypertension, hypothyroidism, and history of subfertility. Investigations were made to look for biochemical markers such as oral glucose tolerance test (OGTT), lipid profile, luteinizing hormone:follicle stimulating hormone (LH:FSH), testosterone, dehydroepiandrosterone sulphate (DHEAS), and anti-Müllerian hormone (AMH). Transvaginal ultrasonography was done to look for the presence of polycystic ovarian morphology on ultrasound or any other associated pelvic disorders. Endometrial biopsy was done for patients presenting with menstrual irregularities to rule out malignancy or hyperplasia. These patients were managed appropriately according to their presenting symptoms and followed up to see the outcome of the treatments given to them. Patients with infertility were investigated and managed with lifestyle modifications, metformin, ovulation induction, laparoscopic ovarian drilling, or in vitro fertilization, as required. Patients with menstrual irregularities were managed by levonorgestrel intrauterine system, hysterectomy, or radical surgery, as required. A note was made of the management and outcomes for all patients.

For statistical analysis, data were collected and presented in terms of percentages and the total number of patients.

## Results

A total of 36 patients fulfilling the criteria for the diagnosis of PCOS during the defined period were included. Among them, 29 were in the age group of 35-40 years, 6 were in the age group of 40-60 years, and 1 patient was above 60, as shown in Table 1.

**Table 1:** Age-wise distribution of patients presenting with polycystic ovarian syndrome (n=36)

Age group (years)	Number of patients (n=36)
35-40	29 (80%)
40-60	6 (16.6%)
>60	1 (2.78%)

As there was only one patient above 60, we divided the remaining patients into two groups: those in the age group of 35-40 years and the other above 40. The different clinical parameters such as BMI, parity, and presenting complaints of the two groups are shown in Table 2.

**Table 2:** Clinical presentation of patients

Clinical parameters	35-40 years (n=29)	>40 years (n=7)
Body mass index >25	21 (72.41%)	5 (71.42%)
Parity - nulliparous	22 (75.86%)	2 (28.57%)
Parity - multiparous	7 (24.13%)	5 (71.42%)
Presenting complaint - infertility	22 (75.86%)	2 (28.57%)
Presenting complaint - menstrual irregularity	07 (24.13%)	5 (71.42%)
Hirsutism present	11 (37.93%)	2 (28.57%)

There were a total of 29 (80.55%) patients in the age group of 35-40, of whom 21 (72.41%) were overweight, 22 (75.86%) were nulliparous and presented with complaints of infertility and 7 (24.13 %) had menstrual irregularity. Hirsutism was present in 11 (37.93%) patients. There were 7 (19.45%) patients in the age group >40 years of whom 5 (71.42%) were overweight. Most of them (5 out of 7 patients, 71.42%) were multiparous and presented mainly with menstrual irregularities (71.42%). Infertility and hirsutism were seen in two patients each (28.57%).

Table 3 shows the comorbidities associated with PCOS patients.

**Table 3:** Associated comorbidities

Distribution of cases as per age	Diabetes mellitus	Hypertension	Hypothyroidism	History of subfertility
35-40 years (n=29)	7 (24.14%)	4 (13.79%)	4 (13.79%)	1 (3.45%)
>40 years (n=7)	2 (28.57%)	2 (28.57%)	3 (42.86%)	2 (28.57%)

Among the 29 patients below 40 years of age, 7 (24.14%) had diabetes mellitus, 4 (13.79%) had hypertension, 4 (13.79%) had hypothyroidism, and 1 (3.45%) had a history of subfertility. In patients above 40 years of age, 2 (28.57%) had diabetes mellitus, 2 (28.57%) had hypertension, 3 (42.86%) had hypothyroidism and 2 (28.57%) had a history of subfertility.

Table 4 shows the biochemical parameters observed in PCOS in aging women.

**Table 4:** Biochemical parameters in women with polycystic ovarian syndrome OGTT, oral glucose tolerance test; LH, luteinizing hormone; FSH, follicle stimulating hormone; DHEAS, dehydroepiandrosterone sulphate; AMH, anti-Müllerian hormone

Biochemical parameters	35-40 years (n=29)	>40 years (n=7)
Impaired OGTT	14 (48.28%)	4 (57.14%)
Impaired lipid profile	4 (13.79%)	2 (28.57%)
Altered LH:FSH	17 (58.62%)	3 (42.86%)
Increased testosterone	7 (24.13%)	0
Increased DHEAS	0	0
Increased AMH (in infertile patients)	10 out of 22 infertile patients (45.45%)	Not examined

Impaired lipid profile was diagnosed if the high-density lipoprotein (HDL) cholesterol level was <40 mg/dl, low-density lipoprotein (LDL) cholesterol level was >140 mg/dl or triglyceride level was >150 mg/dl. Considering the biochemical parameters, it was observed that among women in the age group of 35-40 years, 14 (48.27%) had impaired OGTT, 4 (13.79%) had impaired lipid profile, 17 (58.62%) had altered LH:FSH, and testosterone was increased in 7 (24.13%) subjects. AMH was increased in 10 out of 22 (45.45%) infertile patients in the age group of 35-40 years, whereas in women above 40, 4 (57.14%) had impaired OGTT, 2 (28.57%) had impaired lipid profile, and 3 (42.86%) had altered LH:FSH.

Polycystic ovarian morphology was seen on transvaginal USG in 19 (65.52%) out of 29 patients in the age group of 35-40 and 3 (42.86%) out of 7 patients above 40 years of age. Table 5 shows the presence of polycystic ovaries on transvaginal USG in aging women with PCOS.

**Table 5:** Polycystic ovaries on ultrasonography in women with polycystic ovarian syndrome

Polycystic ovaries on ultrasonography	Present	Absent
35-40 years (n=29)	19 (65.52%)	10 (34.48%)
>40 years (n=7)	3 (42.86%)	4 (57.14%)

A total of 24 patients presented with infertility: 22 in the age group of 35-40 years and 2 above 40 years of age. Table 6 shows the management of and outcome in patients presenting with infertility.

**Table 6:** Management of and outcome in cases presenting with infertility OVI, ovulation induction; LOD, laparoscopic ovarian drilling; IVF, in vitro fertilization

No. of cases (n=24)	Lifestyle modification	Metformin	History of OVI	LOD	OVI/IVF	Outcome
2	+	+	-	-	-	Conceived
4	+	+	+	-	OVI	Conceived
3	+	+	Failed	+	IVF	Conceived
1	+	+	Failed	+	OVI	2 missed abortions
6	+	+	Failed	+	OVI	Conceived
6	+	+	Failed	-	IVF	Conceived
2	+	+	Failed	+	OVI followed by IVF	Conceived

Almost all the infertile patients (23 out of 24, 95.83%), except for one, conceived and carried full-term pregnancies after appropriate management with lifestyle modifications, metformin, laparoscopic ovarian drilling (LOD), ovulation induction (OVI) or in vitro fertilization (IVF). Out of the total 24 infertile patients, 2 (8.33%) conceived after management with only lifestyle modifications and metformin. Another 4 (16.66%) conceived after ovulation induction in addition to lifestyle modification and metformin; 6 (25%) patients opted for in vitro fertilization and conceived along with lifestyle modification and management. Laparoscopic ovarian drilling was done in 12 (50%) patients of whom 11 (91.66%) conceived by OVI or IVF after LOD, whereas one patient had two recurrent missed abortions and was lost to follow-up thereafter.

Table 7 shows the evaluation of cases presenting with irregular cycles.

**Table 7:** Evaluation of cases presenting with irregular cycles EB, endometrial biopsy; HPE, histopathological examination; HMB, heavy menstrual bleeding; LNG-IUS, levonorgestrel intrauterine system; TLH, total laparoscopic hysterectomy; PMB-post menopausal bleeding; RPLND, retroperitoneal pelvic lymph node dissection

S. no.	Presenting complaint (n)	Lifestyle modification	Metformin	EB-HPE	Treatment
1	Oligomenorrhea + HMB (7)	+	+	Secretory (3) and simple hyperplasia (4)	LNG-IUS
2	HMB with fibroid (1)	+	+		TLH
3	PMB (2)	+	+	Endometrial adenocarcinoma	Modified radical hysterectomy with RPLND

A total of 10 out of 36 patients (27.77%) had menstrual irregularities as one of the presenting symptoms. The majority of the patients with irregular cycles had oligomenorrhea combined with heavy menstrual bleeding (70%) and were treated with levonorgestrel intrauterine system (LNG-IUS), as their endometrial biopsy showed histopathological features of secretory endometrium or simple hyperplasia. One patient (10%) had heavy menstrual bleeding with associated fibroid uterus and underwent a hysterectomy, whereas two patients (20%) presenting with post-menopausal bleeding and malignancy had to undergo radical surgery.

## Discussion

This study was conducted to evaluate aging women with PCOS and study their characteristics, symptomatology, biochemical markers, and treatment given, and follow them up to see their treatment outcomes. Over three years, only 36 aging women were diagnosed with PCOS. This must be representing the tip of the iceberg, as these were the patients who had to reach a tertiary care center to seek medical attention for the symptoms. There must no doubt be several undiagnosed or asymptomatic cases for every single case seen. As there are no criteria yet to diagnose PCOS in aging women, the actual numbers will continue to be missed, underdiagnosed, and underreported. We included women over 35, considering them to be aging women, since this is the median age in the lifespan of women, considering an average lifespan of 70 years for Indian women. It was observed that 80% of the women were below 40 years of age, while only 20% were above 40. This could be explained by the age-related amelioration of PCOS symptoms. Furthermore, older PCOS women not having menstrual complaints as their presenting symptom might have presented them to another department of the hospital for their co-morbidities and not to the gynecology department; hence, they might have been missed from being included in our study. A total of 70% of the women were found to be overweight or obese. Infertility was seen in 75% of patients below 40 years of age and 28% in patients above 40, signifying an inability to conceive spontaneously due to anovulation. The main presenting complaint in women below 40 was infertility, which is not surprising, as the socio-cultural factors in this region demand conception, childbearing, and family conception, preferably before 40. However, in women above 40, menstrual irregularities constituted the most common presenting symptom. This might be explained by the prolonged exposure to estrogen due to anovulation.

In our study, we found no significant change in the prevalence of obesity in PCOS women with aging. However, Meun et al. showed an increase in the incidence of obesity and metabolic disorders above age 45 [9]. The prevalence of hirsutism was seen to decline from 37.93% to 28.57%, which is consistent with the findings of Liang et al. who showed that hirsutism prevalence decreases with aging [10]. Diabetes mellitus was seen to increase from 24.14% to 28.57% with aging, similar to the findings of Wang et al. where there was a two-fold increase in the incidence of diabetes mellitus over time [11]. The prevalence of hypertension almost doubled with aging, from 13.79% to 28.57%, which is consistent with the findings of Pinola et al. and the Dallas heart study [12,13]. The prevalence of impaired glucose tolerance and dyslipidemia also increased with age as in other similar studies by Meun et al. and Wang et al. [9,11]. An increase in testosterone and DHEAS levels was seen in 24 % of the women below 40 years of age, vis-à-vis none in the older group, implying a 100% decline with aging. Winters et al. demonstrated 50% lower androgen levels with the increasing age, just as Carmina et al. showed a 25% decrease in androgen levels with the increasing age [14,15]. Barry et al. found a significantly increased risk of endometrial carcinoma in PCOS women with aging, similar to our findings of endometrial carcinoma in two post-menopausal women [16]. Thus, it is clear that the findings of our study are consistent with those of previous studies.

Limitations

This study included patients from the gynecology outpatient department alone, thus missing many aging PCOS patients who may have primarily presented to other departments. Furthermore, as this study consisted of a patient population from a localized region, ethnic variability was not taken care of, which questions the external validity of the study. The limitation of a small sample size is also very obvious. Psychological aspects like depression and mood disorders seen in aging PCOS women were not evaluated. Metabolic disorders increase with age that could be a confounding factor. Further long-term, prospective, large-sized studies are very much required before arriving at any conclusions on PCOS in aging women.

## Conclusions

The aging PCOS population is burdened with multi-morbidity associated with several long-term health risks, which greatly affects the quality of life. This should be considered a public health priority, owing to the health burden and cost implications and the associated low quality of life. Moreover, vast numbers are still missed or unreported due to inadequate diagnostic criteria in this population. Patients presenting with infertility can achieve conception through proper and timely management. Malignancies need to be screened and detected timely. Other associated comorbidities like diabetes, hypertension, impaired glucose tolerance, impaired lipid profile, etc. require adequate medical attention for their control. However, adequate studies to guide proper management in these scenarios are still unavailable. The aging population with PCOS also deserves to live a healthy life without being pulled back due to their disease burden. Therefore, further research in this field is the need of the hour.
